# High hydrostatic pressure induces vigorous flagellar beating in *Chlamydomonas* non-motile mutants lacking the central apparatus

**DOI:** 10.1038/s41598-020-58832-8

**Published:** 2020-02-06

**Authors:** Toshiki Yagi, Masayoshi Nishiyama

**Affiliations:** 1 Department of Life Sciences, Faculty of Life and Environmental Sciences, Prefectural University of Hiroshima, Shobara, Hiroshima 727-0023 Japan; 2 The Hakubi Center for Advanced Research, Kyoto University, Yoshida, Kyoto 606-8501 Japan; 3 Department of Physics, Faculty of Science and Engineering, Kindai University, 3-4-1 Kowakae, Higashiosaka City, Osaka 577-8502 Japan

**Keywords:** Cellular motility, Cilia

## Abstract

The beating of eukaryotic flagella (also called cilia) depends on the sliding movements between microtubules powered by dynein. In cilia/flagella of most organisms, microtubule sliding is regulated by the internal structure of cilia comprising the central pair of microtubules (CP) and radial spokes (RS). *Chlamydomonas paralyzed-flagella* (*pf*) mutants lacking CP or RS are non-motile under physiological conditions. Here, we show that high hydrostatic pressure induces vigorous flagellar beating in *pf* mutants. The beating pattern at 40 MPa was similar to that of wild type at atmospheric pressure. In addition, at 80 MPa, flagella underwent an asymmetric-to-symmetric waveform conversion, similar to the one triggered by an increase in intra-flagella Ca^2+^ concentration during cell’s response to strong light. Thus, our study establishes that neither beating nor waveform conversion of cilia/flagella requires the presence of CP/RS in the axoneme.

## Introduction

**Figure 1:**
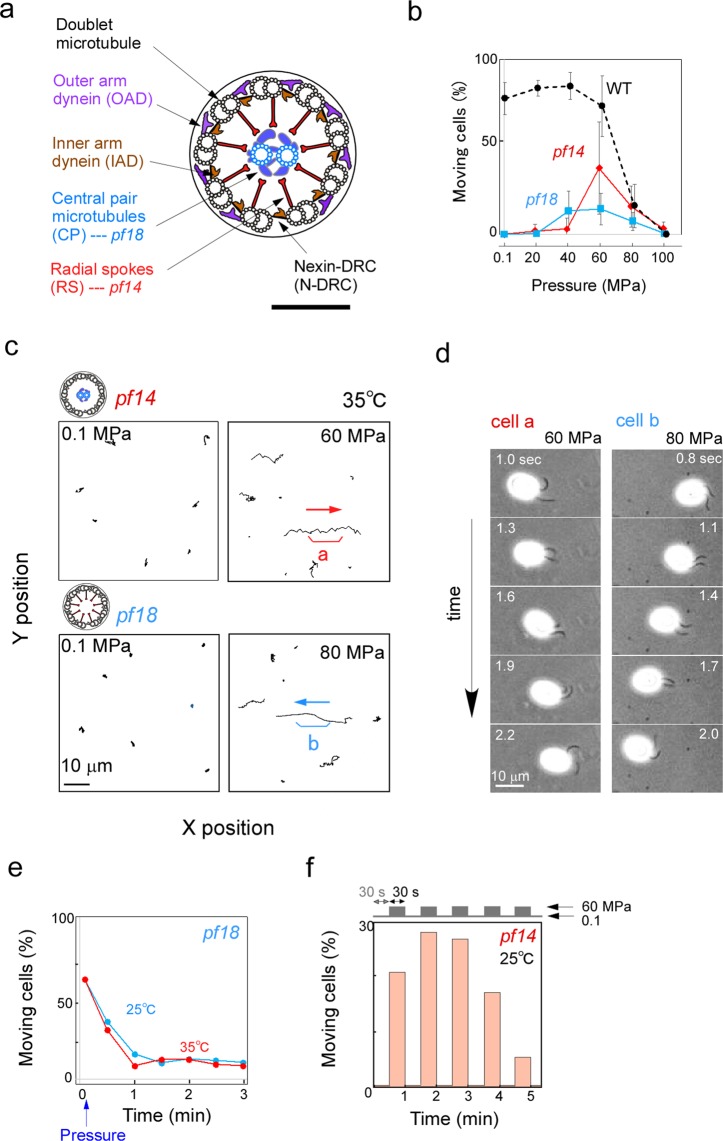
Flagella of *Chlamydomonas* paralyzed-flagella (*pf*) mutants beat at high pressure. (**a**) Schematic illustration of the *Chlamydomonas* flagellum in cross section. Mutant flagella lacking the central pair (CP) (blue) or radial spokes (RS) (red) are paralyzed under physiological conditions. Bar: 100 nm. (**b**) Percentage of motile cells under different pressure conditions. Cells of *pf14*(Red)*, pf18*(blue), and WT (black) were examined. Mean ± SD for more than 20 cells were examined in WT and *pf* mutants, respectively. Temperature: 25 °C. (**c**) Typical swimming trajectories of *pf* cells for three seconds at atmospheric and high pressures. (Upper panel) *pf14* lacking radial spokes. (Lower panel) *pf18* lacking the central pair. At high pressure, a number of cells randomly swam for short distances, while some cells swam straight (cell a and b). Temperature: 35 °C. (**d**) (Left) Asymmetric bending pattern in a forward-swimming cell of *pf14* (cell a in c). Bending of the two flagella is asynchronous. (Right) Symmetric bending pattern in a backward-swimming cell of *pf18* (cell b in c). Bending of the two flagella is synchronous. Cells were observed by high-speed video microscopy. Numbers on the left or right indicate the time after the onset of recording. (**e**) Time course of the *pf18* cell movement after being activated by pressure application. The fraction of moving cells reached a peak within 10 seconds, and then gradually decreased. More than 40 cells were examined for each time point. The time course was similar at 25 and 35 °C. A small percentage of cells continued to swim for more than 3 minutes. (**f**) Reversibility of the pressure-induced motility in *pf14*. A high pressure (60 MPa) and the atmospheric pressure (0.1 MPa) were alternately applied each for 30 sec. The number of moving cells 10 sec after the pressure change was shown in the graph. Cells stopped and resumed movements, both within 1 sec after the release from and application of high pressure, respectively. More than 120 cells were examined for each time point. Temperature: 25 °C.

Cilia and flagella are beating organelles that propel cells through fluids or produce fluid flows over the cell surface. The internal structure of cilia and flagella, the axoneme, has an evolutionally conserved “9 + 2” structure, composed of nine peripheral doublet microtubules and two central microtubules (central pair: CP) (Fig. 1a). The nine outer doublets and the CP interact with each other through radial spokes (RS) projecting from each doublet microtubule. Adjacent doublets are crosslinked by a protein complex called the nexin/dynein regulatory complex (N-DRC)^1^. The outer doublet also attaches inner-arm dynein (IAD) and outer-arm dynein (OAD) projecting toward the adjacent doublet, which drive sliding between outer doublets to produce axonemal beating^2^.

Various lines of evidence indicate that the microtubule sliding is regulated by CP and RS^3^. *Chlamydomonas* mutants lacking the CP or RS are non-motile under physiological conditions, and are called *paralyzed-flagella (pf)* mutants^4,5^. In contrast to the axonemes isolated from wild type (WT), which undergo beating upon addition of ATP, axonemes from *pf* mutants lacking the CP/RS do not beat under the same conditions^5^. However, the flagella and axonemes of *pf* mutants can beat under certain genetic and chemical conditions. First, in the background of a certain mutation (suppressor mutation) in N-DRC, IAD or OAD, the *pf* flagella can beat without recovering the missing structure^6^. This and other observations suggest that N-DRC and CP/RS cooperate in regulating dynein activities in the axoneme^7^. Second, axonemes from *pf* mutants, while unable to beat in the normal reactivation buffer containing physiological concentrations of ATP, can beat in reactivation solutions with low concentrations of ATP or with ATP plus ADP^8^. Regulatory nucleotide binding by dyneins may elicit this phenomenon^9–11^. Axonemes of *pf* mutants can also beat when appropriate concentrations of salts or organic compounds are added to the normal reactivation buffer^12^. The wide range of effective chemicals led to the hypothesis that a change in the solvation of axonemal proteins underlies the beating of the *pf* axonemes^12^. Experiments using various dynein-deficient mutants indicated that OAD, but not IAD, is essential for generation of beating. Thus, the change in solvation or non-physiological nucleotide conditions may produce axonemal beating by modulating the OAD activity.

In the present study we examined the effect of hydrostatic pressure on live *Chlamydomonas* wild type and mutants, and unexpectedly found that *pf* mutants become motile at high pressures. In addition, at higher pressure, cells changed swimming direction from forward to backward by changing the flagellar waveform. Thus, at high pressure, neither flagellar beating nor waveform conversion requires the CP/RS.

## Results and Discussion

### Pressure-induced flagellar beating in *Chlamydomonas* non-motile mutants

Using high-pressure microscopy^13^, we examined the motility of wild type (WT) and two kinds of non-motile *paralyzed-flagella* (*pf*) mutants, *pf18* lacking CP and *pf14* lacking RS (Fig. 1a), at various pressures up to 100 MPa. In WT, the number and speed of swimming cells decreased with increasing pressure (Fig. 1b; Fig. S1). All cells stopped swimming at 100 MPa. Such an inhibitory effect of pressure on the beating of motile cilia and flagella is consistent with previous reports^14,15^. However, the *pf* mutants displayed peculiar responses to high pressure. When the pressure was raised to 80 MPa, many *pf18* and *pf14* cells started to swim within several seconds (Fig. 1c,d, Movie S1). The fraction of moving cells immediately after pressure application was about 60% but decreased to 10% in 3 min (Fig. 1e). When released from high pressure, all cells stopped swimming. Upon re-application of 60 MPa, a fraction of cells started to move again (Fig. 1f). Thus, the pressure-induced motility induction is reversible at least partially.

In a series of analysis under varying hydrostatic pressure (from 0.1 to 100 MPa) and temperature (from 5 to 35 °C), the fraction of the moving cells peaked at lower pressures with decrease in temperature (Fig. S2). This behavior is consistent with the idea that the beating and non-beating states are in equilibrium, and that the equilibrium changes with pressure and temperature; we can think that the *pf* mutants are non-motile under physiological conditions because the equilibrium is somehow shifted to the non-beating state.

**Figure 2:**
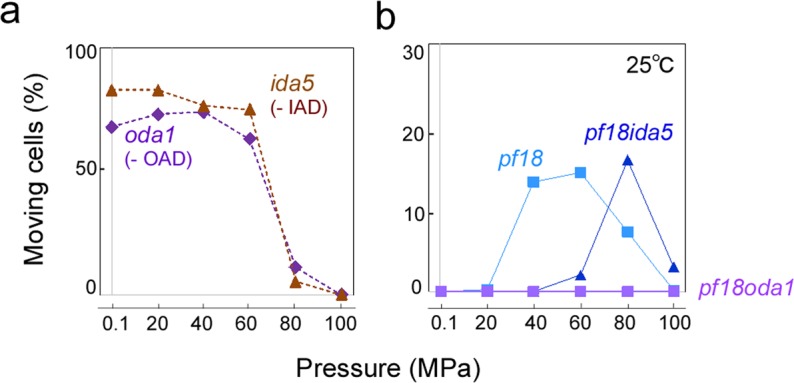
Percentage of moving cells in mutants lacking central pair and different types of dyneins. Motility of (**a**) dynein deficient mutants and (**b**) double mutants lacking central pair and dyneins at high pressure. The mutant *oda1* lacks the entire outer-arm dynein (OAD), whereas the mutant *ida5* lacks several inner-arm dynein species (IAD) (Table S1). The number of moving cells decreased with the increase of pressure in the single dynein mutants, as observed in WT. While *pf18ida5* moved at high pressures like *pf18*, *pf18oda1* displayed no movement at any pressure. More than 20 cells were examined for each data point. Temperature: 25 °C.

Eukaryotic flagella have two types of axonemal dyneins, inner-arm and outer-arm dyneins (IAD and OAD) (Fig. 1a). Previous studies showed that IAD is important for generating strong bending of flagella, while OAD is important for generating high beat frequency^16–18^. To explore the mechanism of pressure-induced activation of *pf* mutants, we investigated the motility of *pf18* cells with the background of *oda1* or *ida5*, mutation causing loss of the entire OAD^17,18^ or several IAD species^16,19^ (Table S1). Like WT, these dynein-deficient mutants gradually decreased their motility with increase in pressure (Fig. 2a). The double mutant *pf18ida5* displayed vigorous flagellar beating at 80 MPa. The optimal pressure for motility induction in *pf18ida5* was higher than in *pf18*, suggesting that some IAD species facilitate the induction of beating in the *pf* mutants at high pressure, although they are not prerequisite for motility. In contrast, *pf18oda1* displayed no movements at any pressure (Fig. 2b; Table S1). Thus, OAD seems to be critical for flagellar beating of *pf18* at high pressure.

The requirement of OAD for pressure-induced flagellar beating in *pf* mutants is reminiscent of previous studies. One study showed that mechanical stimulation of live *pf* mutants induced temporary flagellar beating (oscillation lasted only for <10 cycles), and that OAD was indispensable for this motility^20^. Other studies showed that some of the suppressor mutations that restore flagellar beating in *pf* mutants^6^ have mutations in the β or γ heavy chains (HCs) of OAD^21,22^, suggesting that modulation of these HCs could induce beating of *pf* flagella. Although previous studies have thus observed flagellar beating in *pf* mutants under certain conditions, our present study is the first to show fairly stable flagellar beating in live *pf* mutants without any additional mutation.

**Figure 3:**
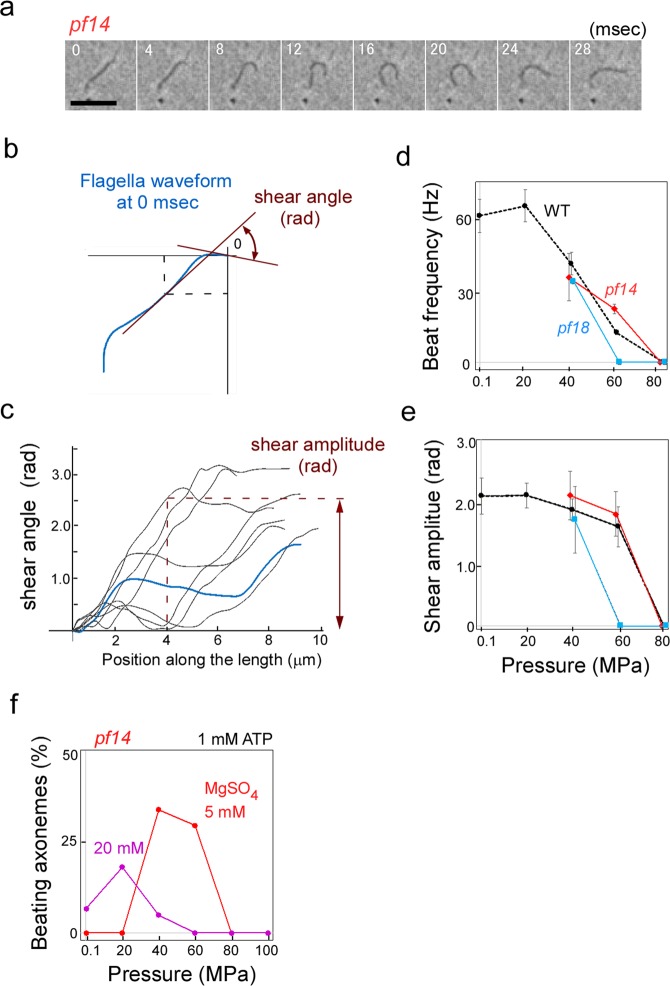
Reactivated motility of axonemes at high pressure. (**a**) Sequential photographs of a *pf14* axoneme beating with an asymmetric waveform in the presence of 1 mM ATP and 1 mM.EGTA at 40 MPa. Temperature: 25 °C (**b**) A trace of waveform in (**a**). The angle between the tangents to the proximal segment (position 0, at upper right position) and multiple positions every 0.25 μm along the axoneme were measured. The angle (shear angle) is proportional to the sliding distance between doublet microtubules at each position^50^. (**c**) The shear angle at every 0.25 μm is plotted along the length of axoneme (shear curve). Shear curves for waveforms at different time points in (**a**) were overlaid; the shear curve at 0 ms is drawn in blue. Shear amplitude at 4 μm from the proximal end (indicated by the dashed line) is used for waveform comparison. (**d**,**e**) Beat frequency and shear amplitude in the beating axonemes of WT and *pf* mutants at different pressures. Mean ± SD were measured in 10 axonemes each for WT and mutants. Temperature: 25 °C. (**f**) Pressure-induced beating of *pf14* axoneme at different MgSO_4_ concentrations. Optimal pressure for *pf14* axonemes were lower at 20 mM MgSO_4_ than at 5 mM MgSO_4_. More than 50 cells were examined for each data point.

The activation of flagellar motility in the *pf* mutants may be brought about through a direct action of pressure on the axoneme. Alternatively, motility may be induced through some vital cellular function. To distinguish between the two possibilities, we performed *in vitro* assays at high pressures using isolated axonemes^23^. In a reactivating buffer containing 1 mM ATP and 1 mM EGTA, at atmospheric pressure, WT axonemes displayed vigorous beating with asymmetric waveform, but *pf14* axonemes displayed no movements^5^. However, application of 40 MPa pressure induced beating in some axonemes (Fig. 3a; Movie S2). The fraction of beating axonemes in the total axonemes reached 10–30% at 40–60 MPa. Similar results were obtained with *pf18* axonemes (Movie S2). These observations strongly suggest that the applied pressure induced flagellar movements in *pf* cells by directly acting on the axoneme.

Almost all (~99%) axonemes of WT and mutants beating in the reactivation buffer displayed asymmetric pattern (Fig. 3a; Movie S2) at any pressure. At 40 MPa, the beat frequency of *pf14* was 35 ± 10 Hz (mean ± SD, *n* = 14), which was about half of the WT frequency at atmospheric pressure, 0.1 MPa (63 ± 7 Hz, *n* = 10) (Fig. 3d). The shear amplitude of *pf14* at 40 MPa was 2.2 ± 0.4 rad (*n* = 14), which was similar to that of WT at 0.1 MPa, 2.1 ± 0.2 rad (*n* = 10) (Fig. 3e). Thus, overall, the beating pattern of *pf* mutant axonemes at 40 MPa is similar to that of WT under physiological conditions.

We previously showed that the presence of ATP plus salts (such as MgSO_4_) or organic compounds induced WT-like beating in *pf* mutant axonemes *in vitro*, and proposed that those chemicals induced axonemal motility by changing protein solvation in the axoneme^12^. In a pressure-application experiment, we found that *pf* axonemes started to beat at lower pressure when the MgSO_4_ concentration was increased from the standard 5 mM to 20 mM (Fig. 3f). High MgSO_4_ concentrations and high pressure are thus apparently additive in the effect to induce motility in *pf* axonemes. Both may cause a perturbation of the protein solvation in the axoneme. In fact, both high pressure and the addition of salt or organic compounds are known to change protein conformation through a change in solvation^24–27^. Since OAD was necessary for motility induction by pressure as well as by salt^12^, we surmise that a change in protein solvation might change the manner of interaction between OAD and doublet microtubules.

The OAD HCs specifically affected by high pressure may be identified by examining the motile properties of isolated OAD HCs and microtubules, using an *in vitro* system similar to the one used to analyze the properties of kinesin under high pressure^28^. Information about specific OAD HC may also be obtained from experiments applying pressure on mutants lacking a specific HC, such as *oda11, oda4-S7*, and *oda2-t*^29–31^.

Although high pressure could directly affect the activity of dynein HCs, naturally it could also affect the hydration and conformation of all axonemal proteins. Their changes may induce large-scale changes in the axoneme, leading to a modulation of dynein activity. For example, a model of axonemal beating mechanism called the Geometric Clutch model postulates that a change in distance between adjacent outer-doublet microtubules switches the dynein-doublet interaction on and off ^32,33^. In accordance with this model, many suppressor mutations, i.e. mutations that restore motility in *pf* mutants, have mutations in N-DRC^34,35^, IAD^36^, or OAD^21,22^, structures that may critically affect the inter-doublet distance. We could imagine that the high hydrostatic pressure, as well as high salts, induces motility in the *pf* mutants by also affecting the inter-doublet distance through a change in protein solvation.

The mechanism that produces oscillatory bending movements in cilia and flagella is still not established even though various models, including Geometric Clutch model, have gained certain experimental supports. Our observations allow us to rule out any hypothesis that postulates an essential role of CP and RS in the generation of axonemal beating.

### Switching of the swimming direction by pressure

**Figure 4:**
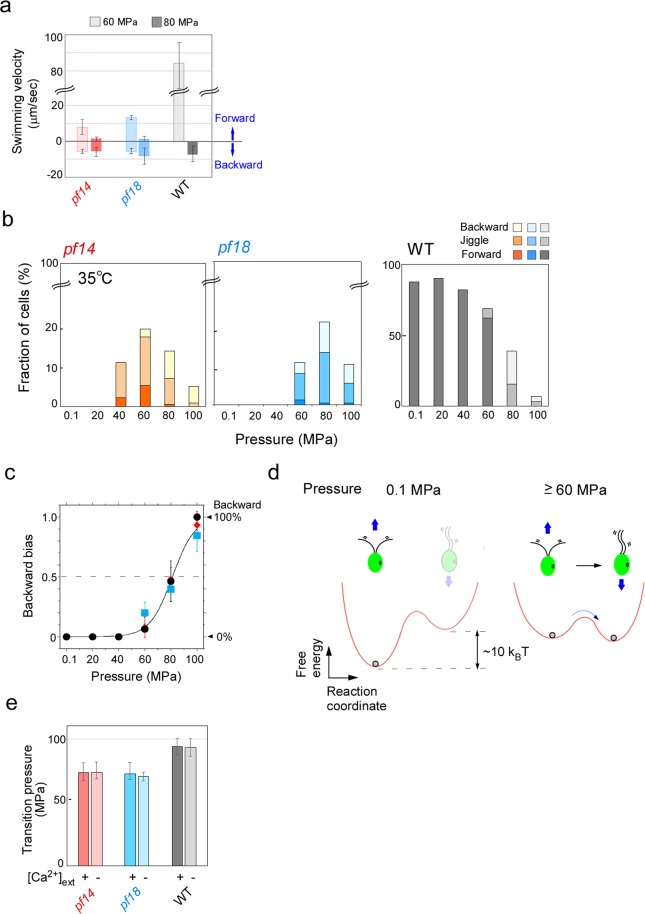
Backward-swimming cells increase at high pressure. (**a**) Comparison of forward- and backward-swimming velocities in WT and *pf* mutant cells. Swimming velocities of more than five cells were measured under respective pressure conditions. Mean ± SD for more than five cells were examined in WT and *pf* mutants, respectively. Temperature: 35 °C. (**b**) The number ratio of forward-swimming, backward-swimming, and jiggling cells under high-pressure conditions for *pf* and WT cells at 35 °C. More than 50 cells were examined for each data point. Similar results were obtained at 15 and 25 °C (Fig. S3). (**c**) Increase in number of backward-swimming cells with the increase of pressure. Backward bias, *n*_*backward*_/(*n*_*forward*_ + *n*_*jiglling*_ + *n*_*backward*_), was calculated, where *n*_*forward*_, *n*_*jiglling*_, *n*_*backward*_ are the numbers of the cells in the forward, jiggling and backward swimming states, respectively. Data for cells whose flagella were stopped were excluded from the calculation. The mean ± SEM of three independent experiments were shown for *pf14* (red), *pf18* (blue), and WT (black). (**d**) Two-state equilibrium model of pressure-induced change of flagellar waveform. The forward-moving (asymmetrically beating) state and backward-moving (symmetrically beating) state would be in the equilibrium. At ambient pressure (0.1 MPa), forward-moving state is prevailing because its free energy potential is lower than that of backward-moving state. High pressure decreases the free-energy potential difference between the two states, and increases the number of backward-moving cells with symmetric beating pattern. Following a two-state model^41^, the backward bias is thermodynamically given by (1 + exp((Δ*G* + *P*Δ*V*)/*k*_*B*_*T*))^−1^, where Δ*G* is standard free energy, *P* is pressure, Δ*V* is a pressure-dependence parameter (reaction volume), *k*_*B*_ is the Boltzman’s constant, and *T* is temperature. The best-fit result was obtained with *ΔG* = 9.4 *k*_*B*_*T*, *ΔV* = −0.49 nm^3^ for *pf* mutant and WT cells (Solid line in c). A gray ball in the left figure indicates that all cells swim forward at atmospheric pressure. In contrast, two gray balls in the right figure indicates that, at high pressure (≥60 MPa), the two states are in equilibrium and that the number of cells moving backward increases with the increase of pressure (arrow). (**e**) The transition pressure (the pressure at which 50% of moving cells displayed backward swimming) for the three strains in the culture medium containing either 0.35 mM Ca^2+^ or 2 mM EGTA. Backward bias with or without Ca^2+^ was analyzed for more than 40 cells, as in Fig. 4b and 4c. The mean ± SEM of the transition pressure in three independent experiments were shown for *pf14* (red), *pf18* (blue), and WT (gray).  All three strains showed almost the same transition pressure in the two media.

In the above experiments, we noticed that some cells under high-pressure conditions swam backward while some swam forward. *Chlamydomonas* cells usually swim forward by beating the two flagella with asymmetric waveforms but, when stimulated by light or other environmental factors, transiently swim backward by changing the waveform to a symmetric pattern^37,38^. As shown in Fig. 1d, forward-swimming *pf* mutant cells displayed an asymmetric flagellar waveform, whereas backward-swimming cells displayed a symmetric waveform (Movie S3). Many other cells displayed jiggling movements such that a cell swam only for a short distance comparative to its body size during recording for a few seconds (Movie S3). WT cells also displayed backward swimming at 80 MPa (Movie S4). The forward swimming velocity of *pf14* cells at 60 MPa was about 10 times slower than the WT velocity at the same pressure, which decreased with increasing pressure (*pf14*, 8.3 ± 4.2 μm/s, WT, 85 ± 14 μm/s; 35 °C) (Fig. 4a). Slow swimming velocity in *pf14* probably resulted from uncoordinated flagellar beating, such as the asymmetric flagellar beating interrupted by a short period of symmetric beating (e.g., Fig. 1d at 1.9 sec), or simultaneous occurrence of two types of waveforms in the two flagella on a single cell (Fig. 1d at 2.2 sec). In contrast, in the backward-swimming mode at 80 MPa*, pf14* cells swam at a velocity comparable to that of WT (*pf14*, 5.7 ± 2.7 μm/s; WT, 7.3 ± 4.1 μm/s; 35 °C) (Fig. 4a). Similar results were obtained with the *pf18* cells (Fig. 4a).

We classified the types of cell movement into forward swimming, backward swimming, jiggling, and non-motile types by eye. As shown in Fig. 4b, at ≤20 MPa and 35 °C, all *pf14* cells were non-motile. At 40–60 MPa, some cells became motile, either swimming forward or jiggling in a small area. At 80–100 MPa, a significant fraction of moving cells swam backward. Similar results were obtained with the *pf18* cells (Fig. 4b). In the case of WT cells, all motile cells swam forward at ≤40 MPa. At higher pressure, some cells displayed backward swimming and the fraction of backward swimming cells increased with pressure (Fig. 4b). At lower temperatures (15 and 25 °C), cells started backward swimming at lower pressure (Fig. S3). To characterize the pressure-induced change in swimming direction, we calculated the probability of motile cells to swim backward (backward bias) by *n*_*backward*_/(*n*_*forward*_ + *n*_*jiglling*_ + *n*_*backward*_), where *n*_*forward*_, *n*_*jiglling*_, *n*_*backward*_ are the numbers of cells in the forward swimming, jiggling and backward swimming states, respectively (Fig. 4c). Non-motile cells were not included in the calculation. The backward bias value increased steeply with the pressure increase, and reached 0.5 at ~80 MPa. Notably, the backward bias increased with pressure similarly for all strains. The observation that flagella can undergo asymmetrical-symmetrical waveform conversion even without CP or RS is consistent with the results reported by a previous study using mutant axonemes reactivated under non-physiological nucleotide conditions at varied Ca^2+^ concentrations^39^.

How might the increased pressure induce waveform conversion? Flagellar waveform conversion in *Chlamydomonas* is known to take place through an increase in intraflagellar concentration of Ca^2+^ from ~10^−7^ to ~10^−4^ M (refs. ^23,40^). Thus, applied pressure might increase intraflagellar concentration of Ca^2+^. Another possibility is that pressure directly modulates some key axoneme proteins to induce waveform change without changing intraflagellar Ca^2+^ concentration. However, the latter possibility is unlikely because, in the absence of Ca^2+^ in the medium, we did not observe any axonemes beating with a symmetrical waveform at high pressure (Fig. 3a).

We hypothesized that the increased Ca^2+^ concentration shifts the equilibrium between the asymmetrically beating state and symmetrically beating state. High pressure could decrease the free-energy potential difference between the two states, which would increase the number of backward-moving cells with symmetric beating pattern (Fig. 4d). Following the two-state model considered for a previous pressure-application experiment^41^, the potential difference was thermodynamically estimated to be ~10 *k*_*B*_*T*, where *k*_*B*_ is the Boltzman’s constant, and *T* is temperature (Fig. 4c,d). The value is close to the chemical potential difference of Ca^2+^ required for flagellar waveform change (refs. ^23,40^), *k*_*B*_*T*ln(10^−7^/10^−4^), which is ~7 *k*_*B*_*T*. This result is thus consistent with the idea that the applied pressure worked to increase the intraflagellar Ca^2+^ concentration.

The light-induced waveform change in live *Chlamydomonas* cells is triggered by Ca^2+^ entry from extracellular medium into flagella through a voltage-gated Ca^2+^ channel^42–44^. We thought that pressure might open the Ca^2+^ channel responsible for the Ca^2+^ entry from the extracellular medium. To test this possibility, we applied pressure in the medium containing 2 mM EGTA instead of the original culture medium that contained 0.35 mM CaCl_2_. To our surprise, significant fractions of *pf14* and *pf18* cells still displayed backward swimming at 80 MPa. The transition pressure at which 50% of moving cells swam backward remained almost the same with and without Ca^2+^ in all strains (Fig. 4e). This result indicates that waveform conversion took place without an influx of Ca^2+^ from extracellular medium. Pressure possibly works on the intracellular Ca^2+^ store^45^ to increase cytosolic Ca^2+^, which then induces flagellar waveform conversion.

Although exactly how hydrostatic pressure induces waveform conversion remains to be studied further, our present study clearly showed that flagella in live *Chlamydomonas* cells can beat without the CP and RS (Fig. 1), and that they can undergo a reversible waveform conversion at high pressure as in WT flagella under physiological conditions (Fig. 4). These observations raise the question regarding the function of CP and RS. It may be much more subtle than generally thought. However, as the flagellar movement of the *pf* mutants at high pressure was vigorous but unstable, we can at least say that one of their functions is to stabilize flagellar oscillatory bending.

## Methods

### Cell strains and culture

*Chlamydomonas reinhardtii* strains 137c (wild type), *oda1* lacking the outer-arm dyenin^17^, *ida5* lacking a subset of the inner-arm dyneins^46^, *pf14* lacking the radial spokes, and *pf18* lacking the central pair^4,5^ were used. Double mutants of the *pf* mutants with *oda1* or with *ida5* were also used. Cells were grown in Tris-acetate-phosphate (TAP) medium^47^ with aeration on a 12 h/12 h, light/dark cycle.

### Reactivation of isolated flagellar axonemes

Flagellar axonemes were prepared by the method of Witman *et al*.^5^ using demembranation of isolated flagella in HMDEK (30 mM HEPES (pH7.4), 5 mM MgSO_4_, 1 mM DTT, 1 mM EGTA, 50 mM K-acetate) containing 0.2% Nonidet P-40 (Nakali tesque, Kyoto, Japan). The demembranated axonemes were reactivated with 1 mM ATP in HMDEKP (HMDEK plus 1% polyethyleneglycol (Mr 20,000; Wako chemicals, Osaka, Japan)).

### High-pressure microscopy

The high-pressure microscope system used has been described elsewhere in detail^13,41^. The sample solution was placed in a high-pressure chamber and mounted on an inverted microscope (Ti–E, Nikon, Tokyo, Japan) equipped with a pressuring apparatus. This apparatus enabled a pressure increase by several tens of MPa within a few seconds. The temperature in the chamber was controlled within ±1 °C^48,49^. Microscopic images were acquired with a CCD camera (WAT–120N^+^, Watec,Tokyo, Japan) at 30 frame s^−1^ or with a high-speed camera (LRH20000B, digimo, Tokyo, Japan) at 500 frames s^−1^. All microscopic images were stored in a computer and analyzed offline using ImageJ software (http://imagej.nih.gov/ij/). *Chlamydomonas* culture medium, TAP medium, contains 0.35 mM CaCl_2_^47^. To investigate the cell motility in the absence of Ca^2+^, Ca^2+^-free TAP medium containing 2 mM EGTA was used.

### Assessment of axoneme motility

The movements of live cells and reactivated axonemes were examined at temperatures between 5 °C and 35 °C, while pressure was being increased from 0.1 to 100 MPa in a stepwise manner.

The beat frequency and bending amplitude of axonemes were assessed by manually-traced bending waveforms. The tangent angle at every 0.25 μm along the axoneme length was measured relative to the angle in the proximal segment (Fig. 3b). A shear curve was obtained by plotting the angles (shear angles) for the total length^50^ (Fig. 3c). Shear curves for specific time points were overlaid, and the angular variation in the shear curves, at a position 4 μm from the base, was regarded as the representative amplitude^39^ (Fig. 3c).

## Supplementary information

Supplemental information.

Supplemental Video 2.

Supplemental Video 3.

Supplemental Video 4.

Supplemental Video 1.
