# Supplementary material for: High hydrostatic pressure induces vigorous flagellar beating in Chlamydomonas non-motile mutants lacking the central apparatus
Source: Sci Rep. 2020 Feb 6;10:2072. doi: 10.1038/s41598-020-58832-8 (PMC7005269; doi:10.1038/s41598-020-58832-8)
Supplement: Supplementary file 1 — Supplemental information. [file 41598_2020_58832_MOESM1_ESM.pdf]

High hydrostatic pressure induces vigorous flagellar beating in *Chlamydomonas* non-motile mutants lacking the central apparatus

Toshiki Yagi[1, *] and Masayoshi Nishiyama [2, 3, *]

1. Department of Life Sciences, Faculty of Life and Environmental Sciences, Prefectural University of Hiroshima, Shobara, Hiroshima, 727-0023, Japan
2. The Hakubi Center for Advanced Research, Kyoto University, Yoshida, Kyoto, 606-8501, Japan
3. Department of Physics, Faculty of Science and Engineering, Kindai University, 3-4-1 Kowakae, Higashiosaka City, Osaka 577-8502, Japan

Supplemental information

Table S1. ***Chlamydomonas*** **strains used in this study**

	Axoneme structures			Beating	
	OAD*	IAD**	CP# / RS+	0.1 MPa	80 MPa
WT	○	○	○	○	○
oda1	×	○	○	○	○
ida5	○	†×	○	○	○
pf18 / pf14	○	○	×	×	○
pf18ida5	○	†×	×	×	○
pf18oda1	×	○	×	×	×

○: at least 10% of the cells displayed flagellar beating; ×: none displayed flagellar beating

*Outer-arm dynein

**Inner-arm dynein

#Central pair microtubule

+Radial spokes

†Missing dynein a, c, d, and e, which are four of the seven major-type of dyneins (dynein a-f)), and DHC11, which is one of the three minor-type dyneins (DHC3, DHC4, and DHC11)[19].

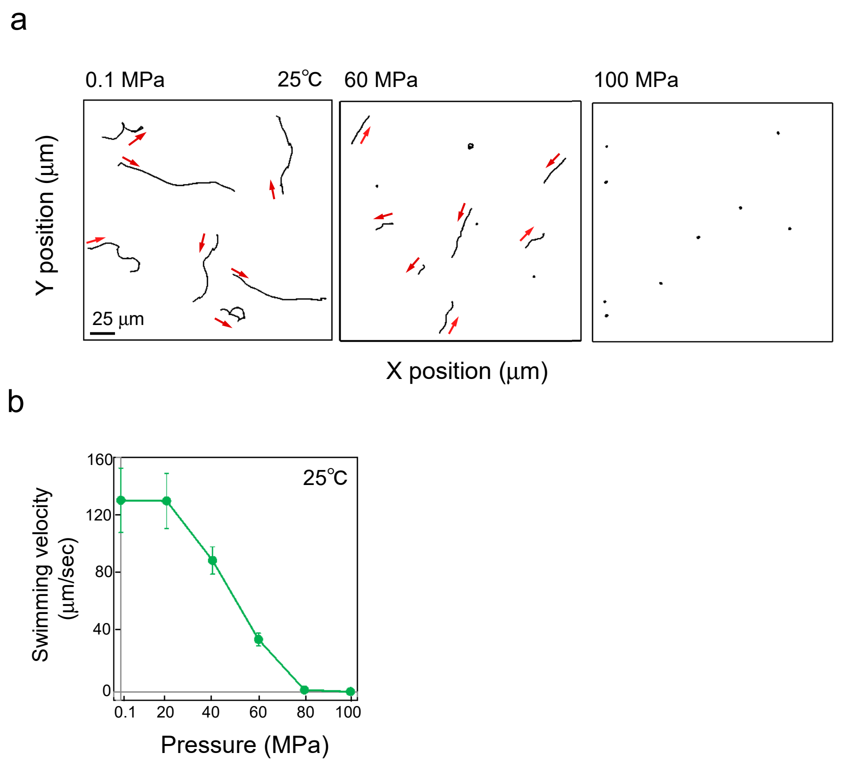

Figure S1. Motility of wild-type cells at high pressure.
(a) Swimming trajectories of WT for one second at 0.1, 60, and 100 MPa. Temperature: 25℃. (b) Swimming velocity of WT cells at various pressures. More than 20 cells were examined for each data point. Temperature: 25℃.

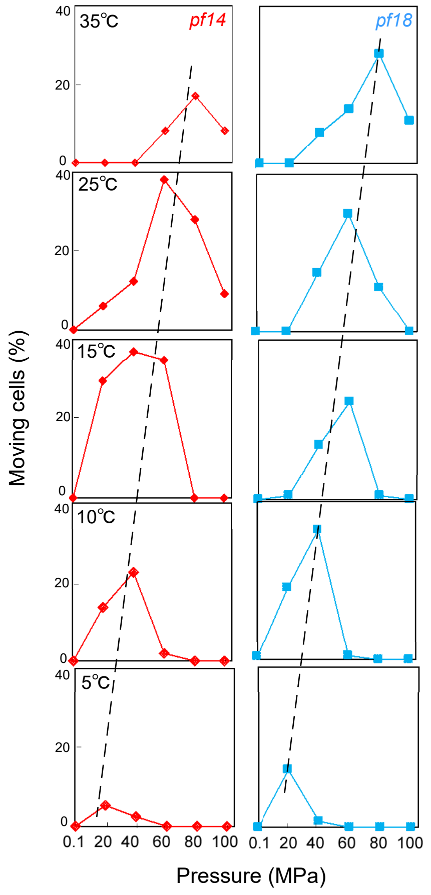

Figure S2. Temperature-dependent changes in *pf14* and *pf18* motility. Percentage of moving cells was calculated under various pressure conditions at 5, 10, 15, 25, and 35℃ (Red, *pf14*; Blue, *pf18*). More than 40 cells were examined for each data point. Broken lines show an almost linear temperature-dependency of the optimal pressure. The lower the temperature, the lower the pressure for motility induction.

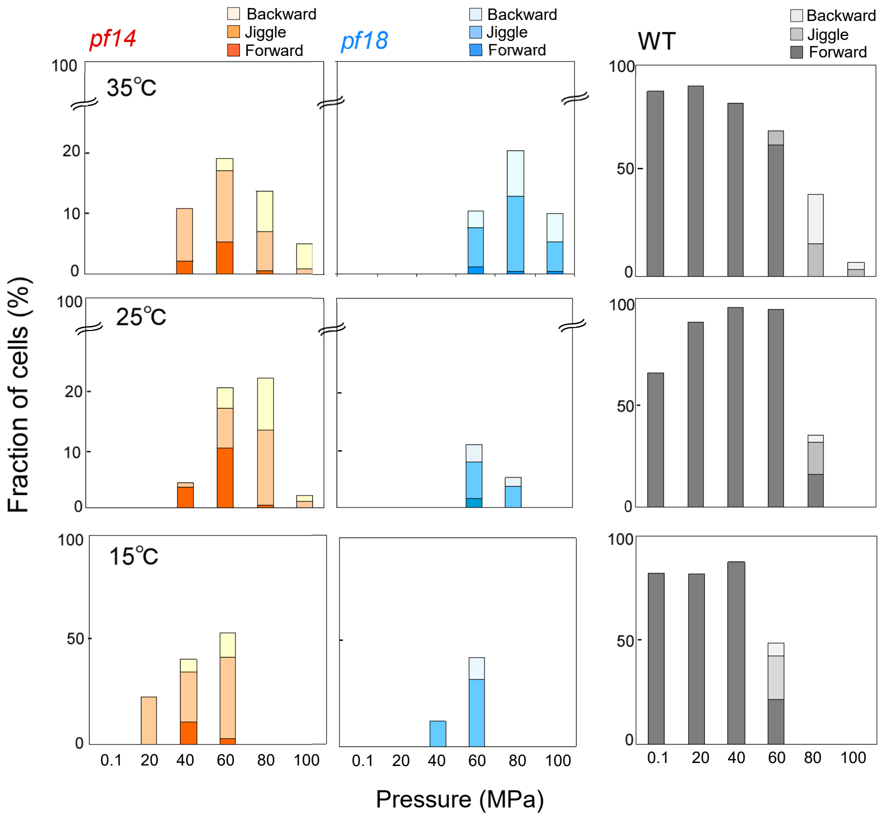

Figure S3. Temperature- and pressure-dependent changes of the moving patterns in *pf* mutant and WT.
The fractions of cells in forward-swimming, backward-swimming, or jiggling states were measured at different temperatures and pressures. More than 40 cells were examined for each data point. At lower temperature, cells started backward swimming at lower pressure.

Movie S1. *pf14* and *pf18* cell motility at ambient (0.1 MPa) and high (60 MPa, 80 MPa) pressures.

Cell movements were recorded with high speed video at 500 frames/sec. The video images are played back at 30 frames/sec. Temperature: 35˚C. Bar: 10 µm.

Movie S2. Bending movements of demembranated and reactivated *pf14* and *pf18* axonemes.

Flagella isolated from cells were demembranated with detergent and the motility of axonemes was reactivated in the presence of 1 mM ATP and 1 mM EGTA at 40 MPa (*pf14*) and 60 MPa (*pf18*). The video images were recorded at 500 frames/sec and are played back at 30 frames/sec. Sequential photographs of the beating *pf14* axoneme were shown in Fig. 3a. Temperature: 25˚C. Bar: 10 µm.

Movie S3. Typical examples of forward-moving, jiggling, and backward-moving cells.

Movements of *pf14* at high pressure recorded at 500 frames/sec and played back at 30 frames/sec. The swimming trajectory of a forward-moving cell is shown as "cell a" in Fig. 1c (*pf14*, at 60MPa), and that of a backward-moving cell as "cell b" in Fig. 1c (*pf18*, at 80MPa). The jiggling-movement was observed at 60 MPa. Note that the two flagella on a single forward-moving *pf14* cell often showed different waveforms. In contrast, most of backward-moving cells showed coordinated symmetric flagellar waveform. Temperature: 35 ˚C.

Movie S4. Symmetrical flagellar beating in WT cells at 80 MPa.

WT cell motility was observed at 80 MPa, 35˚C. The video images were recorded at 500 frames/sec and are played back at 30 frames/sec.
